# Mammalian target of Rapamycin inhibition and mycobacterial survival are uncoupled in murine macrophages

**DOI:** 10.1186/1471-2091-15-4

**Published:** 2014-02-14

**Authors:** Alfred J Zullo, Kristen L Jurcic Smith, Sunhee Lee

**Affiliations:** 1 Human Vaccine Institute and Department of Medicine, Duke University Medical Center, Durham, North Carolina 27710, USA; 2 Yale School of Public Health, Yale University, New Haven Connecticut 06520, USA

**Keywords:** Autophagy, Mycobacteria, mTOR, Inhibitors, Bacille Calmette-Guérin (BCG), *M. tuberculosis*

## Abstract

**Background:**

Autophagy is a cellular response to intracellular pathogens including mycobacteria and is induced by the direct inhibitors of mammalian target of Rapamycin (mTOR), a major negative regulator of autophagy. Autophagy induction by mTOR inhibition (mTOR dependent autophagy), through chemical means or starvation, leads to mycobacterial killing in infected cells. However, previous work by our group has shown that mycobacterial infection of macrophages naturally induces both autophagy and mammalian target of Rapamycin (mTOR) activity (mTOR independent autophagy). In the current work, we further explore the relationship between mTOR activity and mycobacterial killing in macrophages.

**Results:**

While low concentrations of the mTOR inhibitors, Rapamycin, Torin 1, and Torin 2, can effectively reduce or block mTOR activity in response to lipopolysaccharides (LPS) or mycobacteria, higher concentrations (10 uM) are required to observe *Mycobacterium smegmatis* killing. The growth of *M. smegmatis* was also inhibited by high concentrations of Rapamycin in LC3B and ATG5 deficient bone marrow derived macrophages, suggesting that non-autophagic mechanisms might contribute to killing at high doses. Since mycobacterial killing could be observed only at fairly high concentrations of the mTOR inhibitors, exceeding doses necessary to inhibit mTOR, we hypothesized that high doses of Rapamycin, the most commonly utilized mTOR inhibitor for inducing autophagic killing, may exert a direct bactericidal effect on the mycobacteria. Although a short-term treatment of mycobacteria with Rapamycin did not substantially affect mycobacterial growth, a long-term exposure to Rapamycin could impact mycobacterial growth *in vitro* in select species.

**Conclusions:**

This data, coupled with previous work from our laboratory, further indicates that autophagy induction by mTOR inhibition is an artificial means to increase mycobacterial killing and masks more relevant endogenous autophagic biochemistry that needs to be understood.

## Background

The autophagy pathway was first identified as a stress response that allowed cells to survive when nutrients were scarce [1]. Under such conditions, the lack of amino acids and other basic building blocks leads to a reduction in mTOR signaling, a critical sensor of nutrient availability [2]. The absence of mTOR activity induces a reduction in anabolic activities such as protein synthesis, and autophagy is induced to digest unwanted cellular material and liberate building blocks that can be used to sustain survival. The induction of autophagy in response to reduced mTOR signaling due to nutrient stress is considered an mTOR dependent autophagy. More recently, it has been recognized that autophagy is a critical mechanism by which the host can control the growth of intracellular pathogens such as mycobacteria [3]. Recognition of the invading microbe can be achieved through various mechanisms including pattern recognition receptors (NLRs, TLRs, and sequestome-like receptors), inflammatory cytokine signaling, and even antibiotic-mediated pathogen stress [4]–[8]. Infection of cells with various pathogens and mTOR inhibition via Rapamycin or nutrient starvation leads to the isolation of pathogens within autophagosomes via mTOR dependent autophagy. Fusion of the pathogen-containing autophagosome with a lysosome to form the autolysosome results in the direct digestion of the microbe and the liberation of antigenic epitopes used by MHC-I and MHC-II to stimulate adaptive immune responses [9,10]. For example, infection of dendritic cells with mycobacteria followed by treatment with Rapamycin enhances antigen presentation and vaccine efficacy [11]. Moreover, infection of mice lacking ATG5 with *M. tuberculosis*, a protein essential for the processing of LC3B, results in increased bacterial burdens and enhanced inflammatory responses in comparison to ATG5 expressing mice [12]. Thus, it is essential to better understand how mycobacteria may interact with the autophagy pathway so that enhanced strategies can be designed to improve autophagy-mediated killing, minimize the risk of disease, and bolster productive immune responses.

Previous work by our laboratory has documented that mycobacterial infection naturally induces autophagy in RAW264.7 cells [13]. mTOR induction by mycobacterial infection could be blocked by both Rapamycin treatment and nutrient starvation [13]. However, in contrast to autophagy induced by mTOR inhibition (mTOR dependent autophagy), mycobacterial infection simultaneously induces both autophagy and mTOR signaling. This indicates that mycobacteria induce mTOR independent autophagy responses. These unexpected findings now allow for additional investigation of the relationship between mycobacteria, mTOR, and autophagy, which is the basis of our current work. Our data further supports the notion that the use of mTOR inhibition to study mycobacterial killing (mTOR-dependent autophagic killing) is non-physiologic and thus obscures endogenous biochemistry that is critical for understanding and exploiting host-pathogen interactions to favor pathogen clearance.

## Results

### Characterization of mTOR inhibitors

**Figure 1:**
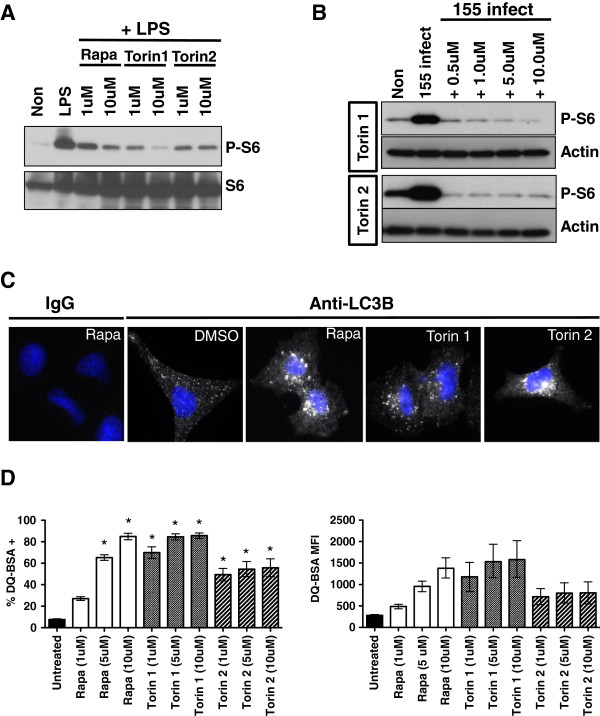
**Low doses of Rapamycin, Torin 1, and Torin 2 inhibit mTOR and induce autophagy. (A)** RAW264.7 cells were pretreated with 1 uM or 10 uM of the mTOR inhibitors indicated and then challenged with 1 ug/ml *E. coli* derived LPS for 3 hours. Protein lysates were prepared and western blots for total ribosomal S6 and phosphorylated ribosomal S6 are shown. Shown are data representative of two independent assays **(B)** RAW264.7 cells were infected with *M. smegmatis* (MOI 5) and treated with the mTOR inhibitors shown. Protein lysates were prepared and western blots for Actin and phosphorylated ribosomal S6 were performed. Shown are data representative of two independent assays **(C)** A549 cells were treated with 10 uM of the indicated inhibitor for 3 hours and then stained for endogenous LC3B, or an isotype control IgG, and imaged by fluorescence microscopy. Shown are data representative of two independent assays. **(D)** RAW264.7 cells were loaded with DQ-BSA, either left untreated (−DMSO) or treated overnight with the indicated concentrations of the mTOR inhibitors shown, and analyzed by flow cytometry. Shown is the combined percentage of DQ-BSA positive cells (+/− SEM) and the mean fluorescent intensity (and intensity range) derived from two independent assays with 3 replicates per assay. For analysis of the percent DQ-BSA positive cells, asterisks indicated p < 0.05 for drug treated samples versus untreated.

Previous studies have demonstrated that mycobacteria naturally induce mTOR activity, as measured by P-S6 induction [13]. Additionally, different species of mycobacteria induce similar levels of P-S6 (mTOR induction), and lipids derived from both *M. smegmatis* and BCG induced similar levels of P-S6 as well. Rapamycin (1 uM-10 uM) inhibits P-S6 induction in response to *Mycobacterium bovis* Bacille Calmette-Guérin (BCG) while 25 uM Rapamycin was used to confirm the ability of *M. smegmatis* to induce mTOR activity. In the current work, we aim to expand upon previous data and further define the connection between mTOR inhibition and mycobacterial killing. A panel of mTOR inhibitors that target mTOR kinase directly (Torin 1 and Torin 2) or indirectly (Rapamycin) was chosen to confirm the capacity of these agents to both inhibit mTOR activity and to induce autophagy [14]. RAW264.7 cells were pre-treated with either 1 uM or 10 uM of each inhibitor and then challenged with 1 ug/ml *Escherichia coli*-derived LPS. All compounds were effective at both 1 uM and 10 uM concentrations to reduce the induction of phosphorylated ribosomal S6 (P-S6), a bona fide mTOR target (Figure 1A). Similarly, Torin 1 and Torin 2 were effective at inhibiting the induction of P-S6 in response to infection with *M. smegmatis* (Figure 1B). Previous studies have shown that Rapamycin can inhibit P-S6 induction in response to mycobacterial infection. Treatment of A549 lung epithelial cells with all compounds elicited robust peri-nuclear LC3B puncta formation, indicating autophagy induction (Figure 1C). A549 cells were chosen to evaluate LCB puncta formation, as they are large cells that readily permit the visualization of endogenous puncta, and they are routinely used to study mycobacterial infection. Lastly, overnight treatment of RAW264.7 cells loaded with DQ-BSA, a self-quenched reporter for proteolysis that correlates well with autophagy [13,15]–[18], indicated that all compounds induce DQ-BSA proteolysis across a wide concentration range (Figure 1D). While 1 uM Rapamycin did not produce statistically significant hydrolysis when compared to untreated cells and higher doses, a response was nonetheless noted. All other concentrations of Rapamycin, and all other inhibitors produced statistically significant DQ-BSA hydrolysis. In sum, we confirm that Rapamycin, Torin 1, and Torin 2 inhibit mTOR in response to a bacterial stimulus and induce autophagy. All three compounds are thus suitable for exploring the impact of mTOR inhibition on mycobacterial survival.

### Higher concentrations of mTOR inhibitors are required for *M. smegmatis* killing

**Figure 2:**
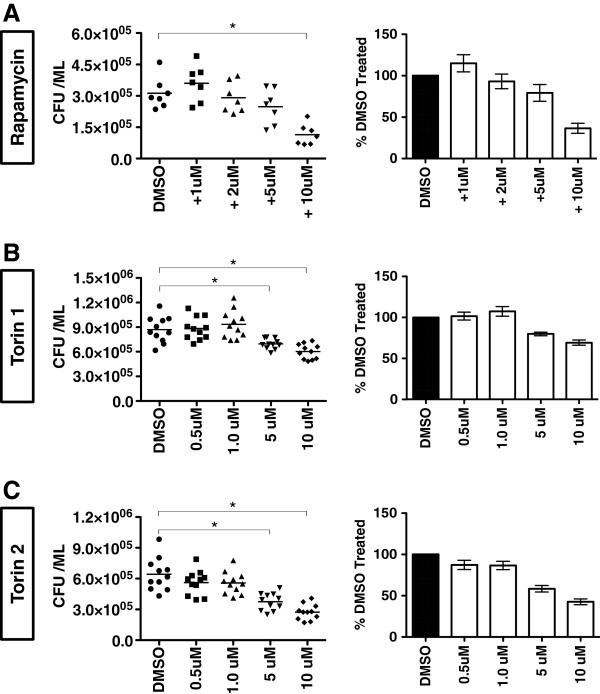
**Low doses of Rapamycin, Torin 1, and Torin 2 are insufficient to kill *M. smegmatis.*** RAW264.7 cells were infected with *M. smegmatis* and treated with Rapamycin **(A)**, Torin 1 **(B)**, or Torin 2 **(C)**. Cells were then lysed and plated for CFU determination. Shown are combined results of two independent assays performed with each inhibitor. On the left are the raw CFU values for each replicate. On the right is the percentage change (+/− SEM) from the mean value of cells treated with DMSO that was set at 100%. For the comparison of raw CFU values, asterisks indicate p ≤ 0.05 for drug treated groups versus DMSO treated cells.

Since as little as 1 uM of each inhibitor was sufficient to demonstrate mTOR inhibition and DQ-BSA hydrolysis in RAW264.7 cells, we evaluated whether equally low concentrations of mTOR inhibitors could produce observable levels of mycobacterial killing. RAW264.7 cells were infected with *M. smegmatis* and treated with the indicated mTOR inhibitors at the concentrations shown. *M. smegmatis* was chosen for these assays as this species naturally induces substantial autophagic responses and thus might be more sensitive to the additive effects of low dose treatment with mTOR inhibitors [13]. While 1 uM of each inhibitor can effectively reduce mTOR signaling, this dose was insufficient to elicit observable *M. smegmatis* killing (Figure 2). Instead, a concentration of 10 uM was required for a significant loss of *M. smegmatis* viability for all three inhibitors in this assay (5 uM dosing produced significant killing in only 2 of 3 compounds tested). As a result, we identify 10 uM as the minimum dose required for robust mycobacterial killing. Lastly, while not used as extensively as Rapamycin, Torin 1, and Torin 2, preliminary work with KU0063794, an additional mTOR inhibitor [19], has thus far revealed similar mTOR inhibitory properties and mycobacterial killing capability (data not shown). In sum, lower doses of Rapamycin, Torin 1, and Torin 2 were insufficient to kill *M. smegmatis,* yet the same doses were effective at reducing mTOR activity.

### Higher concentrations of Rapamycin induce *M. smegmatis* killing in LC3B and ATG5 deficient macrophages

**Figure 3:**
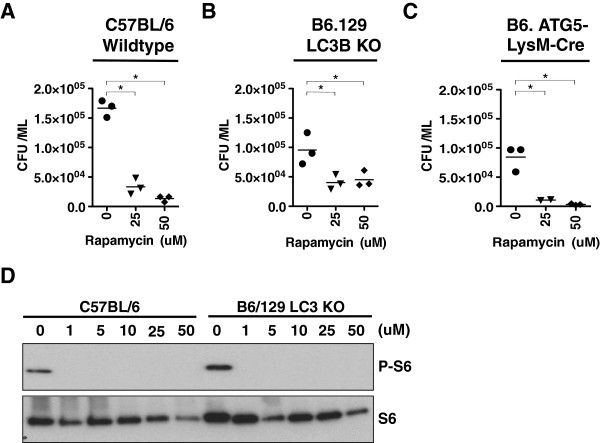
**High doses of Rapamycin can kill *M. smegmatis *in wildtype and autophagy deficient macrophages. (A)** Bone marrow derived macrophages from C57BL/6 mice were infected with *M. smegmatis* and treated with the indicated concentrations of Rapamycin. The cells were lysed and CFU was determined as described in Figure 2. **(B-C)** Bone marrow derived macrophages from B6.129 LC3KO mice and B6 LysM-ATG5 mice were isolated and treated as described above. For parts **(A-C)**, shown are results representative of two independent assays per mouse strain. **(D)** Western blot of protein lysates of samples described in **(A)** and **(B)** for ribosomal S6 and phosphorylated ribosomal S6. Asterisks indicate p ≤ 0.05 drug treated groups versus DMSO treated cells.

Knowing that higher doses of mTOR inhibitors (10 uM or greater) were essential to produce consistent and robust *M. smegmatis* killing, we wanted to determine if the mycobacterial killing induced by higher doses of mTOR inhibition was autophagy specific or due to an unrecognized secondary effect. Similar killing assays were performed in LC3B and ATG5 [20,21] deficient macrophages. Rapamycin was chosen for these assays, as it is the most widely used mTOR inhibitor and its mycobacterial killing properties was comparable to the Torin compounds. We began by first testing C57BL/6 BMDMs to confirm successful differentiation of wildtype macrophages capable of killing *M. smegmatis*. As shown in Figure 3A, both 25 uM and 50 uM of Rapamycin could successfully induce killing in infected C57BL/6 macrophages. Concentrations of 25 uM and 50 uM were chosen based on the observation that at least 10 uM Rapamycin is required for consistent and robust killing in RAW264.7 cells as shown in Figure 2. As a result, 25 uM and 50 uM doses were chosen to guarantee *M. smegmatis* killing in these assays. Similar assays were independently applied to LC3B and ATG5 deficient macrophages. It has been established that ATG5 is critical for efficient autophagic responses to mycobacteria, and that LC3B coated vesicles co-localized with mycobacteria to deliver toxic payloads [5,12,22,23]. Therefore, the goal was to determine how treatment with 25 uM and 50 uM Rapamycin would impact bacterial viability in both mouse models. Treatment of LC3B and ATG5 deficient macrophages with 25 uM and 50 uM Rapamycin yielded observable killing in comparison to Rapamycin untreated macrophages (Figure 3B and C). To confirm that the loss of canonical autophagy does not alter mTOR activity in response to *M. smegmatis* or the response to Rapamycin, western blots from infected cells with or without Rapamycin treatment were performed. As expected, LC3B deficient macrophages could induce P-S6 in response to *M. smegmatis*, and P-S6 induction was blocked by Rapamycin (Figure 3D). We conclude that the ability of higher doses of Rapamycin to induce mycobacterial killing is at least LC3B and ATG5 independent and that a deficiency in the canonical autophagy pathway does not alter mTOR signaling in response to mycobacterial infection.

### *M. smegmatis* and BCG are not directly impacted by Rapamycin in the context of a typical autophagy assay

**Figure 4:**
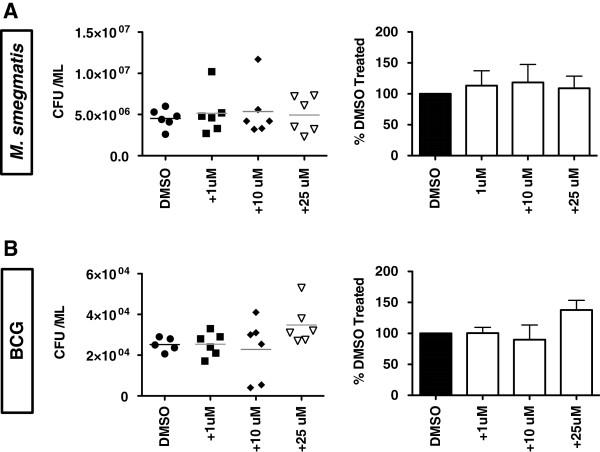
**Rapamycin does not kill *M. smegmatis *or BCG in the absence of macrophages. ***M. smegmatis ***(A)** or BCG **(B)** were cultured in DMEM + DMSO (control) or the indicated doses of Rapamycin. After 3 hours at 37C and 5% CO2, the wells were mixed and the bacteria were plated for CFU determination. On the left are the raw CFU values for each replicate. On the right is the percentage change (+/− SEM) from the mean value of cells treated with DMSO, which was set at 100%. For both **(A)** and **(B)**, shown are the combined results of two independent assays.

The observation that higher of doses of mTOR inhibitors were required to elicit robust mycobacteria killing in RAW264.7 cells led us to suspect that Rapamycin might directly impact mycobacteria in autophagy assays. To test this hypothesis, a modified autophagy assay was performed whereby RAW264.7 cells were eliminated from the assay. *M. smegmatis* and BCG were cultured in DMEM containing 10% HI-FBS under mammalian cell conditions with either DMSO (control) or Rapamycin. After 3 hours, the cultures were harvested and the mycobacteria were plated for CFU determination. As shown in Figure 4A and B, neither *M. smegmatis* nor BCG viability was impacted by Rapamycin in these assays when cultured with up to 25 uM Rapamycin. We conclude that Rapamycin does not appear to directly impact the mycobacteria tested during a standard autophagy assay condition in the absence of the host cells.

### Long-term exposure to Rapamycin can impact the growth of some mycobacterial species

**Figure 5:**
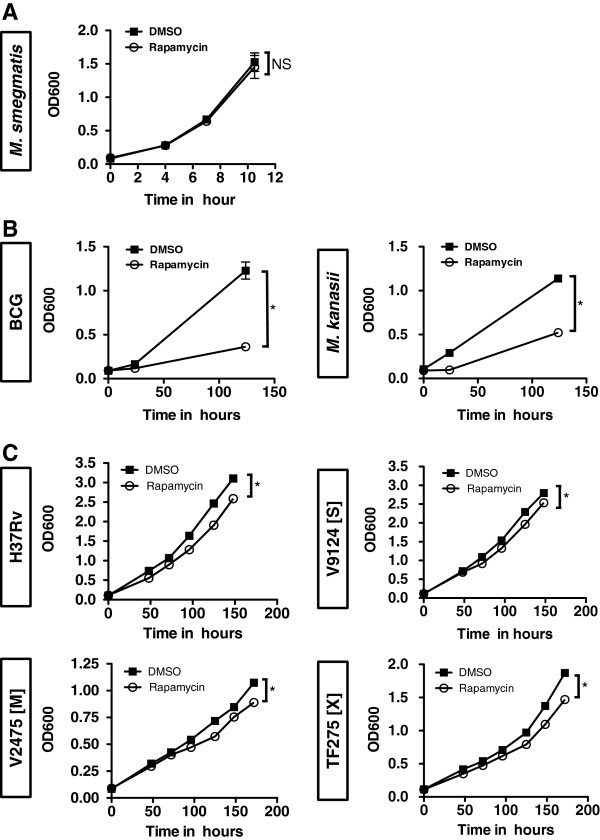
**Long incubations with Rapamycin can alter the growth of some mycobacterial species. (A)** Fast-growing *M. smegmatis* was grown in 7H9 + OADC in the presence of either DMSO (control) or Rapamycin (10 uM). Shown is the OD600 (+/− SEM) at the time intervals described of 7 replicate cultures from 2 independent experiments. The last time point for each growth curve comparing DMSO treatment versus Rapamycin treatment was compared by Student’s T test and found non significant (NS). **(B)** Slow-growing BCG and *M. kanasii* were grown in 7H9 + OADC in the presence of either DMSO (control) or Rapamycin (10 uM). Shown is the OD600 (+/− SEM) at the time intervals described of 10 replicate cultures from 2–3 independent experiments per species. The last time points for each growth curve comparing DMSO treatment versus Rapamycin treatment were compared by Student’s T test. Asterisks indicate p < 0.05. **(C)** The indicated pathogenic mycobacterial species were grown in 7H9-OADC in the presence of DMSO (control) or Rapamycin (10 ug/ml). A laboratory strain (H37Rv), KZN drug-sensitive strain (V9124 [S]), a multidrug resistant (MDR) strain (V2475 [M]), and an extensively drug resistant (XDR) strain (TF275 [X]) were used. Shown is the OD600 (+/− SEM) at the time intervals described of 7–8 replicates from 2 independent experiments. The last time points for each growth curve comparing DMSO treatment versus Rapamycin treatment were compared by Student’s T test. Asterisks indicate p < 0.05.

It has been reported that Rapamycin can attenuate the growth of *Mycobacterium avium* subspecies *paratuberculosis* (MAP) [24]. While the short-term growth of *M. smegmatis* does not appear to be altered by Rapamycin, we wondered if longer exposures of Rapamycin could alter the growth properties of mycobacteria. Several species of mycobacteria were exposed to Rapamycin for longer durations in their typical growth media (7H9-OADC) and the OD600 was monitored for changes in culture growth. *M. smegmatis,* which is a non-pathogenic, fast-growing mycobacterium, was not impacted by Rapamycin through 10 hours of exposure (Figure 5A). In contrast, both BCG and *Mycobacterium kanasii*, which are non-pathogenic, slow-growing mycobacteria, failed to reach an OD600 of 1.0 after over 100 hours of culture (Figure 5B). While somewhat unexpected, the Rapamycin-induced growth inhibition observed in BCG and *M. kanasii* requires incubation periods well beyond that of a standard autophagy assay. Similar assays were performed with *M. tuberculosis* H37Rv, a common laboratory strain, as well as *M. tuberculosis* clinical isolates. While the growth properties of all pathogenic mycobacteria were altered at later time-points (> 100 hours) as indicated by Student’s T test, the magnitude of the differences was modest. There was however no differences observed at shorter time-points (Figure 5C). Thus, it is unlikely that Rapamycin has a direct effect on mycobacterial growth during standard autophagy assays, but it can alter growth after prolonged exposures in some mycobacterial species.

## Discussion

The autophagy pathway has emerged as a versatile cellular mechanism that allows mammalian cells to defend themselves from an array of intracellular microbes [7,9]. Autophagy induction through mTOR inhibition is widely used to demonstrate the autophagic killing of a wide variety of pathogens including mycobacteria. This mTOR dependent autophagy triggers an evolutionarily conserved autophagy response that mimics nutrient deprivation. While extremely effective at inducing pathogen killing, inhibiting mTOR activity may not faithfully recapitulate the biochemistry induced during infection. This is exemplified by previous work from our laboratory demonstrating that mycobacterial infection simultaneously induces both autophagy and mTOR signaling [13]. While largely unexpected, these initial findings now permit additional query into the relationship between mTOR inhibition and pathogen killing.

In the current work, we show that while 1 uM of the mTOR inhibitors Rapamycin, Torin 1, and Torin 2 could reduce mTOR activity, (Figure 1 and previously published), at least 10 uM of the inhibitors were required to demonstrate consistent and significant killing of *M. smegmatis* in RAW264.7 macrophages (Figure 2). This trend of higher concentrations of mTOR inhibitors being required for observable mycobacterial killing appears to exist for both allosteric (Rapamycin) and active site inhibitors (Torin 1 and Torin 2) of mTOR. This is an unexpected result as *M. smegmatis* infection naturally induces substantial autophagy responses such that the kinetic balance should favor killing at lower levels of mTOR inhibition [13]. These findings inspired us to ask if the mycobacterial killing observed with high doses of Rapamycin can occur in macrophages devoid of canonical autophagy components. Using LC3B and ATG5 deficient BMDMs that lack the structural formation of autophagosomes, we showed that 25 uM and 50 uM Rapamycin act through an unappreciated mechanism to induce killing, not through LC3B or ATG5 dependent autophagy (Figure 3). Moreover, a deficiency in the autophagy pathway does not appear to alter mTOR signaling in response to mycobacterial infection. While it is certainly possible that other LC3 or ATG family members, or related signaling downstream of mTOR, could compensate when such a strong stimulus is applied, we must assume that additional autophagic and unidentified cellular responses become involved as the dose of Rapamycin increases. The prospect of additional cellular mechanisms that kill mycobacteria, and are induced upon mTOR inhibition, is an exciting possibility that warrants further investigation.

Since Rapamycin has long been known for its antibiotic properties in fungi and more recently in MAP (*Mycobacterium avium* subspecies *paratuberculosis*) [24], it seemed plausible that Rapamycin may have a direct impact on mycobacteria themselves at higher concentrations. As shown in Figure 4, it is unlikely that within the short time course of a standard autophagy assay that these chemicals could directly interfere with mycobacteria to preclude their viability. The observation that macrophages are required for killing indicates that one or more cellular mechanisms are required for mycobacterial killing under the short time frames and conditions of an autophagy assay. Interestingly, we did observe that certain strains of mycobacteria, such as BCG, *M. kanasii*, and laboratory and clinical isolates of *M. tuberculosis*, did have altered growth properties when exposed to Rapamycin for extended periods of time (Figure 5). While not related to autophagy per se, the data suggests there may be an unrecognized inhibitory target in mycobacteria that exhibits sensitivity to Rapamycin. Additional work in this area will seek to identify this target, utilizing Rapamycin as a foundation, in an effort to design more mycobacterium specific compounds.

Throughout this study we utilized the phosphorylation of ribosomal S6 protein (P-S6) as the indicator of mTOR activity in our assays. While this is a well-known and highly bona fide mTOR target that has been widely utilized as a measure of mTOR activity [13,25], we cannot rule out that other, and perhaps unknown, mTOR targets are better correlates of mycobacterial killing in autophagy assays that utilize mTOR inhibition. This is an exciting idea, since it suggests that there are unrecognized mTOR targets that have a direct connection to mycobacterial infection whose activity is not altered by lower levels of mTOR inhibitors. Proteomics approaches will be required to take an unbiased approach to this question and identify the full spectrum of mTOR targets that are impacted by mycobacterial infection in the presence and absence of various concentrations of mTOR inhibitors.

The use of mTOR inhibitors to induce mTOR dependent autophagic pathogen killing has become the gold-standard assay within the autophagy field. This is somewhat counterintuitive given that nutrient sensing and pathogen sensing utilize unique, and presumably non-overlapping, biochemical mechanisms to affect stimulus specific responses. Taken in sum, our current work strongly suggests that the use of mTOR dependent autophagy to study mycobacterial killing (and possibly other pathogen killing) is artificial and casts shadows on the endogenous host-pathogen biochemistry that naturally occurs during infection. This is consistent with our previous studies indicating that mycobacteria induce mTOR independent autophagy during infection. Future efforts on our part will continue to study mycobacterial autophagy in the absence of artificial influences/inducers to identify specific biochemical events that can be exploited to bolster host defenses. This could be accomplished by a number of methodologies including: proteomics approaches that identify specific post-translational modifications induced shortly after mycobacterial infection; the identification of mycobacterial transposon mutants that are susceptible to macrophage autophagy; the continued use of newly created mouse model systems that are more or less susceptible to mycobacterial infection; and the identification of pharmacological agents that induce autophagy and mycobacterial killing without inhibiting the immunologically sensitive mTOR pathway. Lastly, while mTOR inhibition does carry with it substantial global effects on cellular metabolism, it can not be overlooked that finely tuned mTOR inhibition, especially if restricted to macrophages (alveolar for example), could provide a valuable means to favor host defense against mycobacterial infection.

## Conclusions

While low doses of several mTOR inhibitors are sufficient to reduce mTOR signaling as measured by a reduction in phosphorylated ribosomal S6, the same doses of these compounds are incapable of eliciting robust killing of *M. smegmatis*. In contrast, high doses of Rapamycin, the most common mTOR inhibitor used in autophagy research, induces substantial *M. smegmatis* killing in wildtype macrophages and macrophages from autophagy deficient mice. As it does not appear that Rapamycin has a direct effect on mycobacteria in the short time frames of standard autophagy assays, it suggests that high dose inhibition of mTOR may be acting through an unappreciated cellular mechanism to elicit killing activity. When combined with our previous studies demonstrating that mycobacterial infection naturally induces both autophagy and mTOR signaling, this data reinforces the idea that mTOR inhibition through drugs or starvation is an artificial means of studying mycobacterial killing. We contend that the use of mTOR inhibition to study the molecular mechanisms of host-pathogen interactions is masking the relevant biochemistry that needs to be understood and exploited to favor host defense. However, additional studies further examining the connection between mycobacteria, the mTOR pathway, and host defense need to be performed, as fine-tuning mTOR activity to favor host defense without additional effects would be advantageous and could be developed as a valuable therapeutic.

## Methods

### Mice

Wildtype and LC3B knockout mice [20,21] were purchased from Jackson laboratories. LysM-ATG5 mice were a generous gift from Herbert Virgin (Washington University). All mice were housed in the Duke Human Vaccine Institute Regional Biocontainment laboratory in accordance with institutional animal care and use guidelines.

### mTOR inhibitors

Rapamycin (Sigma), Torin 1, and Torin 2, (Tocris) were dissolved in DMSO to a concentration of 10 mM, aliquoted, and stored at −20°C. Inhibitors were diluted fresh in culture media immediately before use. Lipopolysacharride (LPS) was purchased from Sigma, dissolved in DMEM, aliquoted, and stored at −20°C.

### Mycobacteria

*Mycobacterium bovis* Bacille Calmette-Guérin (BCG) and *M. smegmatis* have been described previously [13]. This study also uses a KZN drug-sensitive strain (V9124 [S]), a multidrug resistant (MDR) strain (V2475 [M]), and an extensively drug resistant (XDR) strain (TF275 [X]). All KZN strains were recovered from patients in KwaZulu-Natal province, South Africa [26]. Unless otherwise noted, mycobacterial strains were cultured in 7H9 media containing 0.5% glycerol, 0.05% tyloxapol, and 10% OADC (Oleic Acid, Albumin, Dextrose, Catalase supplement; hereinafter termed “7H9-OADC”).

### Cell culture, infection, and mycobacterial survival

Murine RAW264.7 macrophages have been described previously [13]. Cells were cultured in DMEM supplemented with 10% heat-inactivated fetal bovine serum (HI-FBS), L-glutamine, sodium pyruvate, and non-essential amino acids. Human A549 alveolar epithelial cells were cultured in RPMI 1640 supplemented with 10% FBS, 1% sodium pyruvate, 1% HEPES and 1% of both non-essential and essential amino acids. For infections, mycobacteria growing in 7H9-OADC were washed in PBS with 0.05% tyloxapol, sonicated to minimize bacterial clumping, and adjusted to the multiplicity of infection 5 (MOI-5). RAW264.7 cells were first infected with mycobacteria, chemical inhibitors were added for the indicated periods of time, and CFU was then determined [13]. For assays involving bone marrow derived macrophages (BMDM), the bone marrow was isolated and depleted of red blood cells. The cells were then differentiated toward the macrophage lineage with DMEM media supplemented with L929 derived culture supernatant [27]. Infection and CFU determination were performed as described above.

### Western blots

The following antibodies were used in this study: anti-Actin plus anti-mouse HRP were purchased from GenScript and Anti-phospho-S6, anti-S6, and anti-rabbit-HRP were purchased from Cell Signaling. Blotting conditions and chemiluminescence have been described [13].

### DQ-BSA assays and flow cytometry

RAW264.7 macrophages were loaded with DQ-BSA as described previously [13]. After an overnight treatment with mTOR inhibitors, DQ-BSA was detected utilizing the PE channels of a BD FACSCanto or a BD LSRII flow cytometer. Flow cytometry was performed at both the Duke University Shared Cytometry Resource and the Yale School of Medicine Cell Sorter Facility. The data were analyzed with FlowJo software.

### LC3 Immunofluorescent staining

A549 cells were cultured on glass coverslips, fixed with 4% formalin, stained with either rabbit anti-LC3B (Cell Signaling) or isotype control, and visualized with Alexa Fluor-594 conjugated anti-rabbit secondary antibodies as described [28]. Counter-staining with Hoechst, fluorescent microscopy, and image analysis were performed as described previously [13,28].

### Statistics

Analysis was performed with GraphPad Prism using an analysis of variance (ANOVA) with a Tukey post-test or Students T test. P values of p ≤ 0.05 were considered to be significant.

## Competing interests

The authors declare that they have no competing interests.

## Authors’ contributions

AZ and KS designed assays, performed experiments, and analyzed data. SL aided in assay design and data analysis. AZ, KS, and SL contributed to the writing and editing of the manuscript. All authors read and approved the final manuscript.
